# Targeted Delivery of Personalized Cancer Vaccines Based on Antibody–Antigen Complexes

**DOI:** 10.3390/vaccines13030324

**Published:** 2025-03-19

**Authors:** Yaling Zhang, Lingling Yan, He Sun, Ziyi Zhang, Fengyun Shen, Lele Sun

**Affiliations:** 1 School of Life Sciences, Shanghai University, Shanghai 200444, China2173474871@shu.edu.cn (L.Y.);; 2 Institute of Materiobiology, Department of Chemistry, College of Science, Shanghai University, Shanghai 200444, China

**Keywords:** neoantigen, cancer vaccine, immunotherapy, antibody–antigen complexes

## Abstract

Background: Personalized cancer vaccines based on tumor neoantigens show great potential in cancer immunotherapy due to their high safety and specificity. However, it is inherently difficult to realize the efficiently targeted delivery of personalized cancer vaccines to antigen-presenting cells (APCs). Methods: This study aimed to address these challenges by developing and evaluating a personalized cancer vaccine based on antibody–antigen complexes, which was designed to enhance antitumor effects by increasing the utilization of tumor neoantigens by APCs. Mice were immunized with a carrier protein, keyhole limpet hemocyanin (KLH), to induce the production of antibodies against KLH. Subsequently, mice were immunized with KLH loaded with tumor neoantigens and the immunoadjuvant CpG ODN and underwent immunological analysis to evaluate the immune and antitumor effects. Results: The results showed that preimmunization with KLH could promote the uptake of the personalized KLH-based tumor vaccine, which was enhanced by dendritic cells (DCs) and macrophages (Mφs), by strengthening the T-cell immune responses to tumors. Conclusions: Collectively, this work provides a new idea for the targeted delivery of personalized cancer vaccines.

## 1. Introduction

Cancer immunotherapy, including CAR-T, bispecific antibody therapy, and cancer vaccine therapy, involves activating the immune system of the body to prevent the spread and metastasis of tumors [1,2]. Among them, cancer vaccine therapy increases the infiltration of lymphocytes into the tumor microenvironment through exposing the immune system to tumor-specific antigens, thereby preventing tumor growth, spread, and recurrence [3,4,5,6], which is considered as a safer cancer immunotherapy strategy. In recent years, personalized cancer vaccines based on tumor neoantigens have been widely applied in cancer immunotherapy due to their multi-target, high safety, and broad-spectrum activity, as well as specific induction of cytotoxic T lymphocytes [7,8,9]. However, due to the inherent instability and poor pharmacokinetics of peptides, efficiently delivering neoantigen peptides to antigen-presenting cells (APCs) is key to activating a specific antitumor T-cell immune response. Although various carriers for delivering tumor neoantigen peptides have been developed, including lipid nanoparticles (LNPs), polymer nanoparticles, bacterial vectors, and multifunctional gel carriers [10,11,12,13], targeted delivery to APCs still faces challenges. First, the immunogenicity of tumor neoantigen peptides is weak, requiring modification or conjugation with other immunostimulants to enhance their immunogenicity [9,14,15,16,17,18]. Second, the delivery efficiency of tumor neoantigen peptides is low, and insufficient processing by APCs limits antigen-specific T-cell immune responses [10].

Studies have shown that the combination of immune complexes and IgG can increase tumor neoantigen uptake by immune cells, including dendritic cells (DCs), macrophages (Mφs), NK cells, and B cells [19]. Therefore, the uptake of antigens in the form of antigen–antibody complexes by DCs can enhance the cross-presentation of antigens, thereby effectively activating CD8^+^ cytotoxic T-lymphocyte (CTL) responses [20,21]. Additionally, Mφs can also improve the utilization of antigens in the form of antigen–antibody complexes [22]. Therefore, utilizing the antigen–antibody complexes formed in the body may help improve the availability of neoantigen peptides in tumors.

**Scheme 1:**
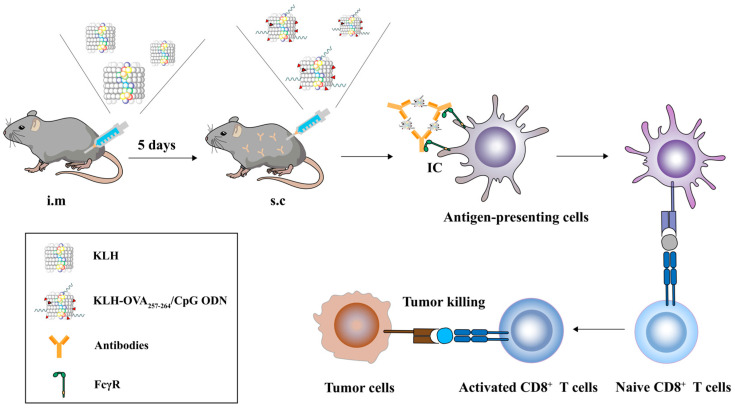
This schematic diagram illustrates the targeted delivery of neoantigen peptides to APCs with a personalized cancer vaccine based on antibody–antigen complexes, activating CD8^+^ T cells to produce an antitumor immune response.

In this work, we immunized mice with a carrier protein, KLH, to induce the production of antibodies against KLH. Then, we used KLH loaded with tumor neoantigens and the immunoadjuvant CpG ODN to immunize mice, utilizing antibody–KLH complexes to achieve the targeted delivery of tumor neoantigens to APCs in vivo. We found that these antibody–KLH complexes can enhance uptake by APCs and Mφs, boost T-cell immune responses, and effectively treat tumors (Scheme 1). Thus, our antigen delivery strategy targeting APCs based on antigen–antibody complexes provides a new approach for the development of efficient personalized cancer vaccines.

## 2. Materials and Methods

### 2.1. Preparation of Tested Samples

#### 2.1.1. Preparation of KLH-OVA_257__–__264_

KLH (Beijing Solab Technology Co., Ltd., Beijing, China) was dissolved in PBS to 10 mg/mL, and sulfo-Lc-SPDP (Thermo Fisher Scientific, Waltham, MA, USA) was dissolved in DMSO (Beijing Bailingwei Technology Co., Ltd., Beijing, China) to 10 mg/mL. A mixture of 100 μL of 10 mg/mL KLH and 2 μL pf 10 mg/mL Sulfo-Lc-SPDP was incubated at room temperature under shaking at 35 rpm for 1.5 h. Subsequently, ultrafiltration was performed at 3000× *g* for 10 min, which was repeated three times. The concentration of pyridyldithiol-activated KLH was measured with the BCA method and found to be 0.765 μM. OVA_257–264_ was dissolved in ultrapure water to 1 mg/mL. Then, pyridyldithiol-activated KLH and OVA_257–264_ (GenScript Biotech Corporation, Nanjing, China) were mixed at a molar ratio of 1:172, resulting in final concentrations of 0.153 μM and 26.316 μM, respectively, and then incubated at 4 °C for 24 h. Pyridyldithiol-activated KLH at 0.153 μM served as a control and was incubated at 4 °C for 24 h.

#### 2.1.2. Preparation of KLH-CpG ODN

Thiol-modified CpG ODN (Shenggong Bioengineering (Shanghai) Co., Ltd., Shanghai, China) was dissolved in ultrapure water to 100 μM, and an excess of TCEP (Shenggong Bioengineering (Shanghai) Co., Ltd.) at 100 times the molar ratio was added, followed by incubation at room temperature under shaking at 85 rpm for 1 h. Subsequently, sodium acetate (Shenggong Bioengineering (Shanghai) Co., Ltd.) at 3 M with a volume equal to 1/8 of the total mixture volume and anhydrous ethanol (Sinopharm Chemical Reagent Shanghai Co., Ltd., Shanghai, China) at a volume four times the total mixture volume were added, mixed well, and then placed at −20 °C for 20 min. After that, centrifugation was performed at 4 °C and 10,000× *g* for 15 min. The supernatant was gently aspirated and discarded, followed by the addition of 200 μL of anhydrous ethanol, which was vortexed to mix and centrifuged at 4 °C, 10,000× *g* for 15 min, which was repeated four times. The supernatant was gently discarded, and, after air-drying, the pellet was dissolved in ultrapure water. Pyridyldithiol-activated KLH and reduced CpG ODN were mixed at a molar ratio of 1:250, resulting in final concentrations of 0.153 μM and 38.25 μM, respectively, which were then incubated at 4 °C for 24 h. Pyridyldithiol-activated KLH at 0.153 μM served as a control and was incubated at 4 °C for 24 h.

#### 2.1.3. Preparation of KLH-OVA_257–264_/CpG

Pyridyldithiol-activated KLH, OVA_257–264_, and CpG ODN were mixed in a molar ratio of 1:160:20, resulting in final concentrations of 0.153 μM, 24.48 μM, and 3.06 μM, respectively, which were incubated at 4 °C for 24 h. Pyridyldithiol-activated KLH at 0.153 μM served as a control and was incubated at 4 °C for 24 h.

#### 2.1.4. Preparation of KLH-AF647

Pyridyldithiol-activated KLH and Alexa Fluor™ 647 NHS ester (Shenggong Bioengineering (Shanghai) Co., Ltd.) were mixed in a molar ratio of 1:20, resulting in final concentrations of 0.36 μM and 7.2 μM, respectively, and incubated under shaking at 100 rpm for 30 min. After dilution with PBS, the mixture was transferred into a 100 kDa ultrafiltration tube and centrifuged at 3000× *g* for 10 min, which was repeated three times to remove excess Alexa Fluor™ 647 NHS ester (Thermo Fisher Scientific, Waltham, MA, USA), and then the KLH-AF647 in the inner tube was collected.

### 2.2. Ultraviolet Detection of Samples

The ultraviolet–visible absorption spectra of pyridyldithiol-activated KLH or KLH-OVA_257–264_, KLH-CpG ODN, and KLH-OVA_257–264_/CpG were characterized with a UV–Vis spectrophotometer (Shanghai Yuanxi Instrument Co., Ltd., Shanghai, China, UV-5500PC). A microcuvette was filled with 100 μL of PBS, and the scanning wavelength range was set from 260 to 430 nm for baseline correction. After washing the microcuvette, 100 μL of pyridyldithiol-activated KLH or KLH-OVA_257–264_, KLH-CpG ODN, and KLH-OVA_257–264_/CpG were added, and the ultraviolet–visible absorption spectra were scanned and recorded.

### 2.3. Antibody Titer Determination

Peripheral blood was collected from mice with or without KLH immunization using the retro-orbital venous plexus puncture method, with 100 μL collected from each mouse. The blood was then left at room temperature for 1 h to clot, followed by centrifugation at 4 °C and 10,000 rpm for 10 min. The supernatant was collected for analysis. KLH at 1 mg/mL was diluted in coating buffer to a final concentration of 2 μg/mL, with 100 μL added to each well and incubated overnight at 4 °C. After washing three times with PBST, the wells were blocked with 1% protein-free blocking solution and incubated at room temperature for 1 h. The wells were washed three times with PBST. Mouse serum was diluted with 1% protein-free blocking solution at 128-fold, 256-fold, 512-fold, 1024-fold, 2048-fold, 4096-fold, 8192-fold, and 16,384-fold dilutions. Then, 100 µL of each diluted serum was added to the wells. The plates were incubated at room temperature for 2 h. After washing three times with PBST, 100 μL of horseradish peroxidase-conjugated goat anti-mouse IgG (Shanghai Biyun Tian Biotechnology Co., Ltd., Shanghai, China) diluted 1:1000 was added and incubated at room temperature for 1 h. The wells were washed four times with PBST (Shenggong Bioengineering (Shanghai) Co., Ltd.). Then, 100 μL of TMB substrate solution (Thermo Fisher Scientific) was added to each well, and the peroxidase reaction was observed at room temperature for 20 min. The reaction was stopped by adding 50 μL of ELISA stopping solution (Shenggong Bioengineering (Shanghai) Co., Ltd.) to each well. The optical density (OD) values were measured at 450 nm and 620 nm with a multifunctional microplate reader (Molecular Devices Corporation, Shanghai, China, SpectraMax iD5). The ratio of the OD values of the KLH-immunized group to the non-immunized group was calculated. A ratio greater than 2 was considered positive, and less than 2 was considered negative. The highest positive dilution ratio was taken as the antibody titer.

### 2.4. Cellular Uptake

To assess the targeting effect of antibody–antigen complexes on the APCs and Mφs, first, 6 μL of 1 mg/mL Alexa Fluor 647-labeled personalized tumor vaccine was incubated with 10 μL of serum for 30 min at 37 °C. Concurrently, as a control, the Alexa Fluor 647-labeled personalized tumor vaccine was incubated with serum from non-immunized mice under the same conditions. BALB/c mice were euthanized. Their spleens were collected, ground in PBS buffer, and centrifuged at 1300 rpm for 3 min. The supernatant was gently discarded, and 1 mL of red blood cell lysis buffer (Shenggong Bioengineering (Shanghai) Co., Ltd.) was added, vortexed to mix, and then centrifuged at 1300 rpm for 3 min. The supernatant was gently discarded, and the cells were resuspended in 1 mL of PBS. The cells were filtered through sterile gauze to collect a uniform cell suspension. The cell count was determined using a hemocytometer. Cells were added to a 96-well transparent round-bottom microplate at a concentration of 1 million per well, resuspended in 200 μL of culture medium. The serum and Alexa Fluor 647-labeled personalized tumor vaccine mixture were added to the cells and incubated at 37 °C for 6 h. CD11c^+^ DCs were stained with FITC anti-mouse CD11c antibody (Biolegend, San Diego, CA, USA) and analyzed using flow cytometry. F4/80^+^ Mφs were stained with FITC anti-mouse F4/80 antibody (Biolegend) and analyzed using a flow cytometer (Beckman Kurt Trading (China) Co., Ltd. Beijing Branch, Beijing, China). Results were analyzed with FlowJo 10.10.

### 2.5. Immune Activation

C57BL/6 mice were randomly divided into 3 groups. First, 50 μg of KLH was injected intramuscularly or not injected. After 7 days, OT-1 mice were euthanized, their spleens was harvested and ground, and red blood cells were lysed to prepare a cell suspension, which was then stained with CFSE (MedChemExpress, NJ, USA). The stained splenocytes were injected via the tail vein into C57BL/6 mice at a dose of 1 × 10^7^ per mouse. Two days later, the experimental group was immunized subcutaneously with KLH-OVA_257–264_/CpG, containing 1 μg of OVA_257–264_ per mouse. Three days later, C57BL/6 mice were euthanized. The spleen was harvested and ground, and red blood cells were lysed to prepare a cell suspension. CD11c^+^ DCs were stained with FITC anti-mouse CD11c antibody (Biolegend) and analyzed with flow cytometry. Results were analyzed with FlowJo 10.10.

### 2.6. Cell Culture

The tumor model was established using B16-OVA cells. The cells were cultured in Dulbecco’s modified Eagle’s medium (DMEM, Shenggong Bioengineering Co., Ltd.) supplemented with 10% fetal bovine serum (FBS, Bovogen Biological Company, Melbourne, Australia), 0.05 × 10^−3^ M 2-mercaptoethanol, 1× penicillin–streptomycin solution, and 1% glutamine (Gln, Shenggong Bioengineering Co., Ltd., Beijing, China). Cultures were maintained in a 5% CO_2_ atmosphere at 37 °C.

### 2.7. Tumor Treatment

For tumor immunotherapy, we firstly established a B16-OVA tumor model in C57BL/6 mice. C57BL/6 mouse were inoculated by subcutaneous injection of 1.5 × 10^6^ B16-OVA cells in 50 μL of PBS onto the back of each mouse. Then, 20 C57BL/6 tumor-bearing mice were randomly divided into five groups. One day after tumor inoculation, the KLH + KLH-OVA_257–26__4_/CpG group was injected with 50 μg KLH intramuscularly. Seven days after tumor inoculation, 5 groups of treatments were created: 1. untreated; 2. KLH treatment; 3. CpG + OVA_257–264_ treatment; 4. KLH-OVA_257–264_/CpG treatment; 5. KLH-OVA_257–264_/CpG treatment (KLH preimmunized). Firstly, the fluorescently labeled OVA_257–264_/H2K^b^ dimer was prepared. Simply, 50 μL of 0.5 mg/mL MHC-I dimer (Dimer X, Becton, Dickinson and Company, Franklin Lakes, NJ, USA, cat. 551323) purchased from BD was mixed with 20-fold excess of NHS-alexa647. After one hour incubation at 4 °C, alexa64-labeled MHC-I dimer was incubated with OVA_257–264_ peptides for 8 h at 4 °C. Then, the excess alexa647 and peptides were depleted using 3 times of ultrafiltration with 100 KDa ultrafiltration tube. The concentration of the prepared OVA_257–264_/H2K^b^ dimer-alexa647 was measured with a protein BCA quantitation kit. For the detection of antigen-specific CD8^+^ T cells, the peripheral blood of the mice was obtained, followed by depleting the red blood cells with red blood cell lysis buffer. Then, the white cells were stained with FITC-labeled CD3 antibody, Percp-labeled CD8 antibody, and OVA_257–264_/H2K^b^ dimer-alexa647. After that, the percentage of antigen-specific CD8^+^ T cell in peripheral blood CD8^+^ T cells was detected with flow cytometry (BD FACScalibur, Becton, Dickinson and Company).

### 2.8. Animal Declaration

Female C57BL/6 or BALB/c mice, aged six weeks and weighing 18–20 g, were obtained fromChangzhou Cavenss Experimental Animals Co., Ltd. ( Changzhou, China), for the animal studies. All animal experiments adhered to the guidelines for the use and care of laboratory animals in biomedical research as set forth by the National Institutes of Health (NIH) (No. 85–23, revised 1996) and were approved by the Ethics Committee for Animal Experiments at Shanghai University (No. 2022-065).

### 2.9. Statistical Analysis

All results are presented as mean ± standard error of the mean (SEM). Statistical analysis was conducted using the two-tailed Student’s *t* test to compare independent groups, with statistical significance set at *p* < 0.05 (indicated by *), *p* < 0.01 (**), and *p* < 0.001 (***).

## 3. Results

### 3.1. Preparation and Characterization of Personalized Cancer Vaccines

**Figure 1:**
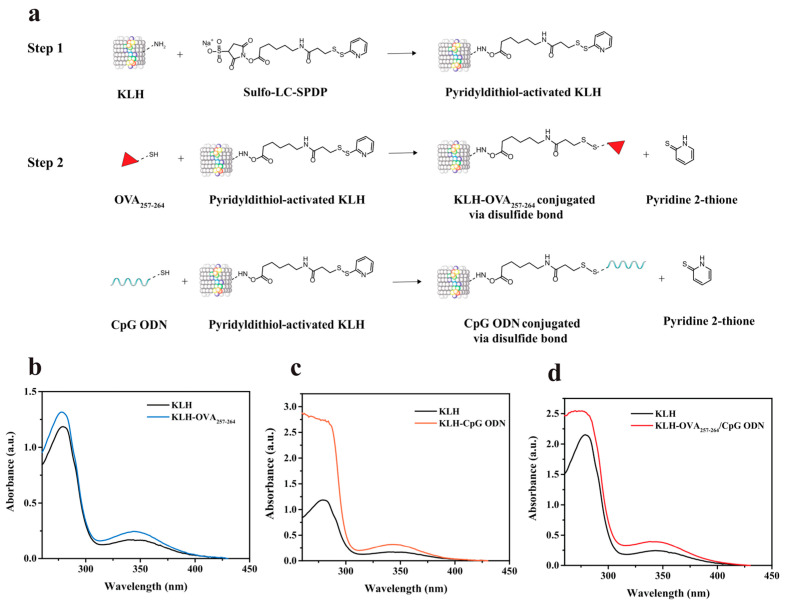
Synthesis and characterization of personalized KLH-based cancer vaccines. (**a**) Schematic diagram showing the conjugation of KLH and OVA_257–264_/CpG ODN using sulfo-LC-SPDP. (**b**–**d**) Ultraviolet–visible (UV–vis) absorption spectrum of the sample solutions after the reaction.

Keyhole limpet hemocyanin (KLH) can induce a strong humoral immune response. In this work, we chose KLH as a carrier protein and peptide OVA_257–264_ as a model that mimics tumor neoantigen to construct a personalized cancer vaccine. As shown in Figure 1a, in order to conjugate the model neoantigen peptide OVA_257–264_ and the immunoadjuvant CpG ODN to KLH, firstly, we mixed KLH with an excess of sulfo-LC-SPDP, a heterobifunctional crosslinker, based on the rapid reaction of sulfo-NHS ester with a primary amine molecule to form pyridyldithiol-activated KLH. Then, thiol-modified OVA_257–264_ and immunoadjuvant CpG ODN were conjugated to the surface of KLH through a reaction with pyridyl disulfide, producing 2-mercaptopyridine with absorbance at 343 nm. To demonstrate that thiol-modified OVA_257–264_ and immunoadjuvant CpG ODN could be conjugated to KLH separately, we first mixed pyridyldithiol-activated KLH with thiol-modified OVA_257–264_ or immunoadjuvant CpG ODN in a molar ratio of 1:200, which we incubated at 4 °C for 24 h before measuring absorbance at 343 nm. As a control, we also incubated pyridyldithiol-activated KLH at the same concentration at 4 °C for 24 h and then measured absorbance at 343 nm.

As shown in Figure 1b,c, the absorbance at 343 nm significantly increased for the sample solutions after the reaction of pyridyldithiol-activated KLH with thiol-modified OVA_257–264_ or immunoadjuvant CpG ODN. Since the conjugation of one-molecule OVA_257–264_ or one-molecule immunoadjuvant CpG ODN to KLH produces one-molecule 2-mercaptopyridine (Figure 1a), by calculating the molar concentration of the newly formed 2-mercaptopyridine in the sample solution and based on the molar concentration of KLH, we estimated that approximately 52-molecule OVA_257–264_ or 104-molecule CpG ODN could be conjugated to 1-molecule KLH. Furthermore, we prepared a personalized vaccine for the B16-OVA tumor model treatment by mixing pyridyldithiol-activated KLH, thiol-modified OVA_257–264_, and thiol-modified immunoadjuvant CpG ODN in a molar ratio of 1:160:20. Based on the net increase in absorbance at 343 nm of the vaccine sample solution, we calculated that approximately 105 molecules of OVA_257–264_ and CpG were conjugated to 1 molecule of KLH.

### 3.2. Preimmunization with KLH Can Promote the Uptake of Personalized KLH-Based Tumor Vaccines by APCs

**Figure 2:**
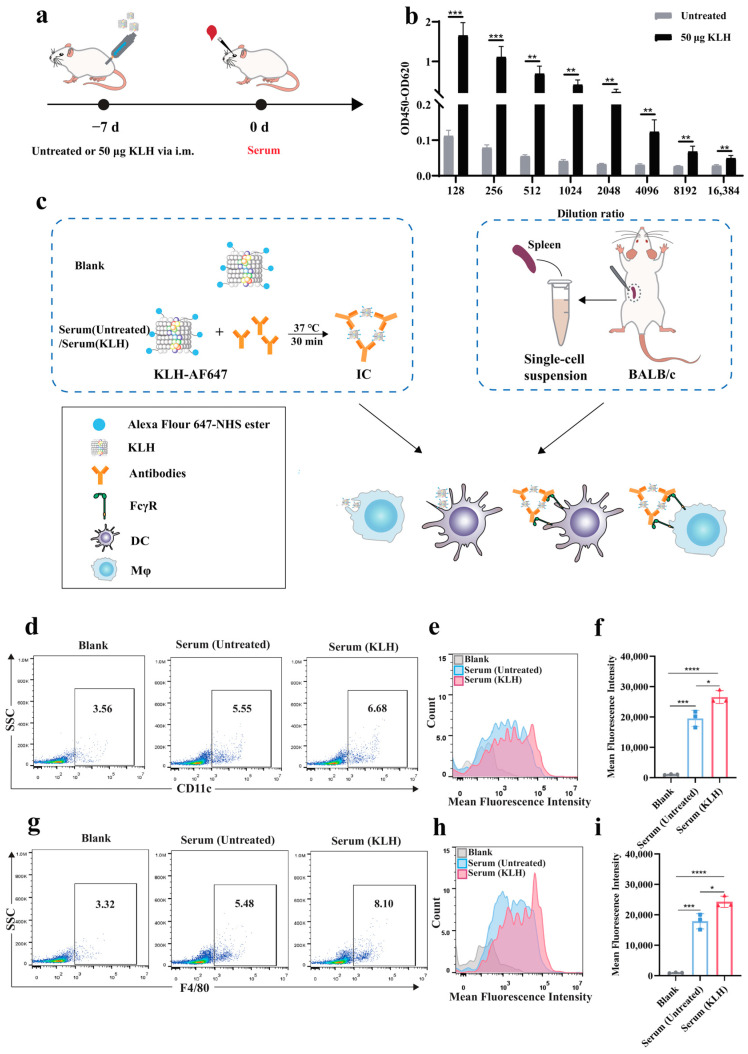
Antibody–KLH complexes enhance the targeting of APCs and Mφs. (**a**) The timeline for preimmunization of BALB/c mice with KLH. (**b**) ELISA detection of anti-KLH antibody titers. Data are shown as mean ± SD, n = 4. (**c**) Schematic diagram of incubation of BALB/c splenic immune cells with KLH-AF 647 or IC in vitro. (**d**–**f**) Flow cytometry analysis of the uptake of KLH-AF 647 by CD11c^+^ DCs in mouse spleen. Data are shown as mean ± SD, n = 3. (**g**–**i**) Flow cytometry analysis of uptake of KLH-AF 647 by F4/80 Mφs in mouse spleen. Data are shown as mean ± SD, n = 3. The statistical analysis was performed with a *t* test. * *p* < 0.05, ** *p* < 0.01, *** *p* < 0.001, and **** *p* < 0.0001.

We preimmunized mice with an intramuscular injection of 50 μg of KLH (female C57BL/6) and then collected serum one week later to detect the IgG titers against KLH in the serum with an enzyme-linked immunosorbent assay (ELISA) (Figure 2a). As shown in Figure 2b, after a single immunization, the mice produced a high level of IgG titers against KLH (close to 10^4^). Furthermore, we aimed to verify whether the serum from KLH-immunized mice could promote the uptake of the personalized tumor vaccines by DCs and Mφs in vitro.

We first conducted uptake experiments in vitro to verify whether DCs or Mφs took up free CpG ODNs or OVA_257–264_. Alexa Fluor 647-labeled CpG ODNs, OVA_257–264_, and their conjugates with KLH were utilized for the in vitro uptake assay involving DCs (Figure S1a–d) and Mφs (Figure S1e–h). DCs or Mφs hardly took up free CpG ODNs or OVA_257–264_; however, conjugation with the KLH carrier enhanced the uptake of CpG ODNs or OVA_257–264_ by DCs and Mφs (Figure S1). After excluding the effects of free CpG ODN and OVA_257–264_ uptake by DCs and Mφs, only three treatments were needed to evaluate the impact of prior immunization on antigen uptake by DCs or Mφs: untreated, KLH-OVA_257–264_/CpG, and KLH + KLH-OVA_257–264_/CpG.

To this end, we first labeled the aforementioned personalized tumor vaccines with Alexa Fluor 647 NHS ester to facilitate the detection of the uptake of the vaccine by APCs with flow cytometry. Then, 6 μL of 1 mg/mL Alexa Fluor 647-labeled personalized tumor vaccine was incubated with 10 μL of serum for 30 min at 37 °C. Concurrently, as a control, we incubated Alexa Fluor 647-labeled personalized tumor vaccines with serum from nonimmunized mice under the same conditions. Subsequently, we incubated the previously stated mixture with splenocytes from C57BL/6 mice at 37 °C for 6 h. As shown in Figure 2d–i, the flow cytometry results indicated that the uptake of the personalized tumor vaccines treated with the serum from the KLH-immunized mice by DCs (CD11c-positive) and Mφs (F4/80-positive) was significantly increased compared to those treated with the serum from untreated mice. In addition, we found that after the incubation of personalized tumor vaccines treated with the serum from the KLH-immunized mice with splenocytes, the proportion of DCs (CD11c-positive) and Mφs (F4/80-positive) was significantly higher than that of mice not preimmunized with KLH. This may have been because the immune complexes formed by the KLH antibodies and the vaccine had a certain activating effect on the APCs.

### 3.3. Preimmunization with KLH Can Enhance the Immune Effect of Personalized KLH-Based Tumor Vaccines In Vivo

**Figure 3:**
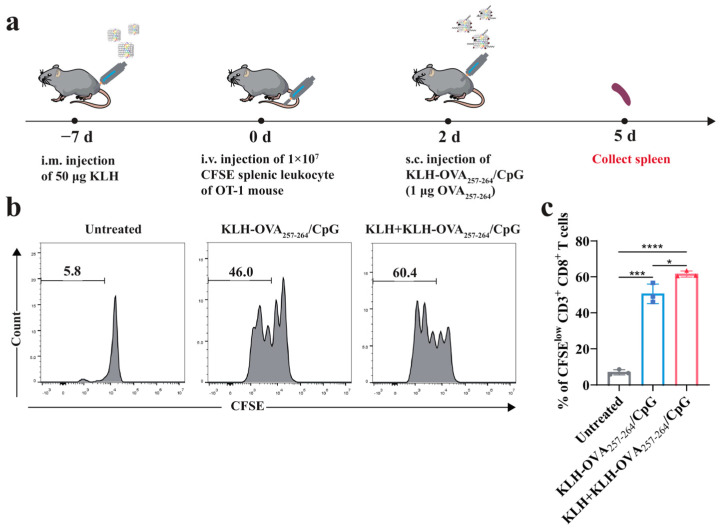
Evaluation of the proliferation effect of CD8^+^ T cells induced by personalized KLH-based tumor vaccines in vivo. (**a**) Timeline for the immunization of C57BL/6 mice. (**b**,**c**) Flow cytometry analysis of proliferation of CFSE-labeled CD8^+^ T cells in vivo. Data are shown as mean ± SD, n = 3. The statistical analysis was performed with the *t* test. * *p* < 0.05, *** *p* < 0.001, and **** *p* < 0.0001.

Next, we assessed whether preimmunization with KLH can enhance the cellular immune response induced by the aforementioned personalized KLH-based tumor vaccine in vivo. Initially, we randomly divided female C57BL/6 mice into three groups, including an untreated group, a KLH-OVA_257–264_/CpG immunization group, and a pre-KLH immunization @ KLH-OVA_257–264_/CpG immunization group (three mice per group). Among these, the pre-KLH immunization @ KLH-OVA_257–264_/CpG immunization group was preimmunized with 50 μg of KLH via intramuscular injection 7 days in advance to induce the production of anti-KLH antibodies (Figure 3a). Subsequently, on day 0, each group received a tail vein injection of 1 × 10^7^ CFSE-stained splenocytes from OT-1 mice. Two days later, the KLH-OVA_257–264_/CpG immunization group and the pre-KLH immunization @ KLH-OVA_257–264_/CpG immunization group were subcutaneously immunized with KLH-OVA_257–264_/CpG (1 μg of OVA_257–264_ per mouse). Three days postimmunization, the mice were euthanized to prepare splenocytes, and the proliferation of CD3^+^ CD8^+^ T cells was analyzed with a flow-cytometry-based CFSE dilution method (Figure 3a). As shown in Figure 3b,c, the untreated mice had only 5.8% CFSElow CD3^+^ CD8^+^ T cells of the total CFSE-labeled CD3^+^ CD8^+^ T cells, the mice immunized with KLH-OVA_257–264_/CpG had 46% CFSElow CD3^+^ CD8^+^ T cells, and the mice preimmunized with KLH and subsequently immunized with KLH-OVA_257–264_/CpG had the highest proportion of CFSElow CD3^+^ CD8^+^ T cells, reaching 60.4%. The result indicates that preimmunization with KLH can significantly enhance the induction of CD8^+^ T-cell proliferation in vivo with the previously mentioned personalized KLH-based tumor vaccine.

### 3.4. Preimmunization with KLH Can Enhance the Antitumor Effects of Personalized KLH-Based Tumor Vaccines

**Figure 4:**
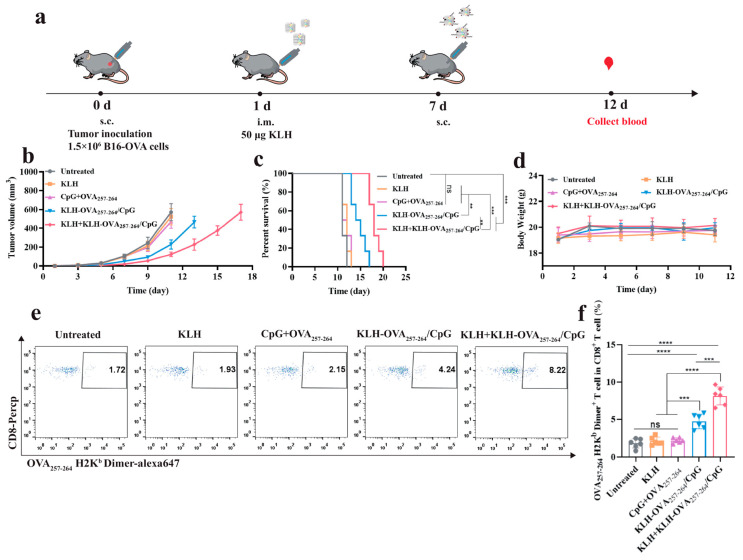
Evaluation of antitumor effects of personalized KLH-based cancer vaccines. (**a**) Treatment timeline for B16-OVA tumor model. (**b**) Tumor growth curves of B16-OVA tumor-bearing mice in different treatment groups. Data are shown as mean ± SD, n = 6. (**c**) Survival curves of B16-OVA tumor-bearing mice under different treatments. Data are shown as mean ± SD, n = 6. (**d**) Body weight of tumor-bearing mice during the observation period. Data are shown as mean ± SD, n = 6. (**e**,**f**) Flow cytometry analysis of activation of OVA_257–264_ H2K^b^ dimer^+^ T cell in vivo. Data are shown as mean ± SD, n = 6. Statistical analysis was performed with log-rank test (**c**) or *t* test (**f**). ns *p*≥ 0.05, ** *p* < 0.01, *** *p* < 0.001, and **** *p* < 0.0001.

Next, a melanoma model was established by subcutaneously injecting 1.5 × 10^6^ B16-OVA cells into female C57BL/6 mice, which were then randomly divided into five groups: an untreated group, a KLH immunization group, a CpG + OVA_257–264_ immunization group, a KLH-OVA_257–264_/CpG immunization group, and a pre-KLH immunization @ KLH-OVA_257–264_/CpG immunization group. As shown in Figure 4a, the mice in the pre-KLH immunization @ KLH-OVA_257–264_/CpG immunization group were immunized with an intramuscular injection of 50 μg of KLH on day 1. Then, on day 7, the KLH immunization group was immunized with 276 μg of KLH per mouse, the CpG + OVA_257–264_ immunization group was immunized with 4.2 μg of CpG and 5 μg of OVA_257–264_ per mouse, and the KLH-OVA_257–264_/CpG immunization group and the pre-KLH immunization @ KLH-OVA_257–264_/CpG immunization group were immunized with 4.2 μg of CpG and 5 μg of OVA_257–264_ (conjugated on 276 μg KLH) per mouse. As shown in Figure 4b, direct immunization with KLH or CpG + OVA_257–264_ had a minimal effect on cancer treatment. However, direct immunization with KLH-OVA_257–264_/CpG significantly slowed the tumor growth in the mice. Interestingly, the mice preimmunized with KLH showed even slower tumor growth (Figure 4b) and extended survival (Figure 4c) compared to those directly immunized with KLH-OVA_257–264_/CpG. Concurrently, the mouse weight monitoring results indicated that our developed personalized cancer vaccine delivery strategy had no significant toxic side effects (Figure 4d). Furthermore, to monitor whether cellular immunity was activated during the above-mentioned treatment, we analyzed the activation of antigen-specific CD8^+^ T cells in the peripheral blood of the mice from each group using flow cytometry on day 12. The results showed that the proportion of OVA_257–264_ H2K^b^ dimer^+^ T cell in the CD8^+^ T cells in the peripheral blood of mice preimmunized with KLH was significantly higher than in the untreated group and those directly immunized with KLH, CpG + OVA_257–264_, or KLH-OVA_257–264_/CpG (Figure 4e,f). This result is consistent with the previously mentioned antitumor effects. Therefore, we speculate that preimmunization with KLH may promote the formation of immune complexes of the proposed personalized cancer vaccine, which are thus more efficiently taken up by APCs, increasing the utilization rate of the neoantigen peptides in vivo, and subsequently inducing stronger antitumor cellular immune responses.

## 4. Discussion

In this study, we leveraged antibody–antigen complexes to enhance the targeted delivery of neoantigen peptides to APCs. This approach shows the efficient and targeted delivery of vaccines to activate a specific antitumor T-cell immune response. Previous studies have established the potential of personalized cancer vaccines based on tumor neoantigens, which are unique to an individual’s tumor and thus offer a high degree of specificity. However, the challenge lies in the delivery of these neoantigens to APCs, which is crucial for initiating an effective immune response. This study explored a novel delivery mechanism in vivo based on previous finding that the engagement of FcγRs on APCs by antibody–antigen complexes can enhance antigen cross-presentation and activate CD8^+^ T-cell responses. This study’s results support and extend these findings by demonstrating a practical application of this mechanism in a personalized vaccine setting.

The observed increase in the uptake of KLH-based vaccines by DCs and macrophages in the presence of pre-existing KLH antibodies suggests that the antibody–antigen complex formation indeed facilitates targeted delivery. The increased proportion of antigen-specific CD8^+^ T cells in CD3^+^ CD8^+^ T cells in preimmunized mice indicates a robust cellular immune response. These findings are significant as they demonstrate the potential of the vaccine to break tolerance and elicit a specific antitumor response. The slowed tumor growth and extended survival in mice preimmunized with KLH and subsequently immunized with the personalized vaccine are promising. These results suggest that the vaccine not only enhances the immune response but also translates to tangible antitumor effects in vivo.

The success of this vaccine strategy in a mouse melanoma model raises the possibility of its application in other cancer types, highlighting the potential for a broad impact on cancer treatment. Currently, immunotherapy based on tumor neoantigens has demonstrated significant potential, with many neoantigens having been identified for human melanoma, glioblastoma, colorectal cancer, and other cancers [23,24]. For example, nine neoantigens associated with human melanoma have been identified [25]. Therefore, the ability to target APCs effectively could revolutionize how personalized vaccines are developed and used in clinical settings. Future studies should focus on optimizing the vaccine formulation to achieve the most significant antitumor effects. This includes determining the optimal ratio of neoantigen to adjuvant and the impact of multiple vaccinations. Investigating the longevity of the immune response induced by the vaccine is crucial. Studies should track the persistence of immune memory and the potential for long-term protection against tumor recurrence. Given the complex nature of cancer, combining this vaccine approach with other immunotherapies, such as checkpoint inhibitors, may yield synergistic effects, which should be further explored.

## 5. Conclusions

In summary, we proposed a cancer vaccine based on neoantigen peptide that targets APCs in vivo with antibody–antigen complexes. This personalized antibody–antigen-complex-based cancer vaccine, with KLH as the carrier protein as well as conjugated to adjuvant CpG ODN and neoantigen peptides, not only has strong immunogenicity but also improves the utilization rate of neoantigen peptides in the body, significantly activating CD8^+^ T cells and enhancing the antitumor immune response. We believe that this antigen–antibody-complex-based strategy for targeting APCs can be applied to various other types of cancer, providing a new approach for the development of effective personalized cancer vaccines.

## Data Availability

All data generated or analyzed during this study are available.

## Supplementary Materials

The following supporting information can be downloaded at: https://www.mdpi.com/article/10.3390/vaccines13030324/s1, Figure S1: Conjugation with KLH carrier can enhance the uptake of CpG or OVA_257–264_ by DCs and Mφs.

## References

[1] Kaur R., Bhardwaj A., Gupta S. (2023). Cancer treatment therapies: Traditional to modern approaches to combat cancers. Mol. Biol. Rep..

[2] Liu C., Yang M., Zhang D., Chen M., Zhu D. (2022). Clinical cancer immunotherapy: Current progress and prospects. Front. Immunol..

[3] Schuster M., Nechansky A., Kircheis R. (2006). Cancer immunotherapy. Biotechnol. J..

[4] Liu N., Xiao X., Zhang Z., Mao C., Wan M., Shen J. (2023). Advances in Cancer Vaccine Research. ACS Biomater. Sci. Eng..

[5] Rui R., Zhou L., He S. (2023). Cancer immunotherapies: Advances and bottlenecks. Front. Immunol..

[6] Schlom J. (2012). Therapeutic cancer vaccines: Current status and moving forward. J. Natl. Cancer Inst..

[7] Thomas S., Prendergast G.C. (2016). Cancer Vaccines: A Brief Overview. Methods Mol. Biol..

[8] Bao Y., Hu Q., Wang X., Feng X., He Y., Guo Y., Fu D. (2020). Chemo-immunotherapy with doxorubicin prodrug and erythrocyte membrane-enveloped polymer nano-vaccine enhances antitumor activity. Biomed. Pharmacother..

[9] Keskin D.B., Anandappa A.J., Sun J., Tirosh I., Mathewson N.D., Li S., Oliveira G., Giobbie-Hurder A., Felt K., Gjini E. (2018). Neoantigen vaccine generates intratumoral T cell responses in phase Ib glioblastoma trial. Nature.

[10] Xu P., Luo H., Kong Y., Lai W.F., Cui L., Zhu X. (2020). Cancer neoantigen: Boosting immunotherapy. Biomed. Pharmacother..

[11] Gong N., Alameh M.G., El-Mayta R., Xue L., Weissman D., Mitchell M.J. (2024). Enhancing in situ cancer vaccines using delivery technologies. Nat. Rev. Drug Discov..

[12] Redenti A., Im J., Redenti B., Li F., Rouanne M., Sheng Z., Sun W., Gurbatri C.R., Huang S., Komaranchath M. (2024). Probiotic neoantigen delivery vectors for precision cancer immunotherapy. Nature.

[13] Shae D., Baljon J.J., Wehbe M., Christov P.P., Becker K.W., Kumar A., Suryadevara N., Carson C.S., Palmer C.R., Knight F.C. (2020). Co-delivery of Peptide Neoantigens and Stimulator of Interferon Genes Agonists Enhances Response to Cancer Vaccines. ACS Nano.

[14] Zhou L., Zhao L., Wang M., Qi X., Zhang X., Song Q., Xue D., Mao M., Zhang Z., Shi J. (2024). Dendritic Cell-Hitchhiking In Vivo for Vaccine Delivery to Lymph Nodes. Adv. Sci..

[15] Hilf N., Kuttruff-Coqui S., Frenzel K., Bukur V., Stevanovic S., Gouttefangeas C., Platten M., Tabatabai G., Dutoit V., van der Burg S.H. (2019). Actively personalized vaccination trial for newly diagnosed glioblastoma. Nature.

[16] Hu Z., Leet D.E., Allesoe R.L., Oliveira G., Li S., Luoma A.M., Liu J., Forman J., Huang T., Iorgulescu J.B. (2021). Personal neoantigen vaccines induce persistent memory T cell responses and epitope spreading in patients with melanoma. Nat. Med..

[17] Katsikis P.D., Ishii K.J., Schliehe C. (2024). Challenges in developing personalized neoantigen cancer vaccines. Nat. Rev. Immunol..

[18] Ott P.A., Hu Z., Keskin D.B., Shukla S.A., Sun J., Bozym D.J., Zhang W., Luoma A., Giobbie-Hurder A., Peter L. (2017). An immunogenic personal neoantigen vaccine for patients with melanoma. Nature.

[19] Ott P.A., Hu-Lieskovan S., Chmielowski B., Govindan R., Naing A., Bhardwaj N., Margolin K., Awad M.M., Hellmann M.D., Lin J.J. (2020). A Phase Ib Trial of Personalized Neoantigen Therapy Plus Anti-PD-1 in Patients with Advanced Melanoma, Non-small Cell Lung Cancer, or Bladder Cancer. Cell.

[20] Galvez-Cancino F., Simpson A.P., Costoya C., Matos I., Qian D., Peggs K.S., Litchfield K., Quezada S.A. (2024). Fcgamma receptors and immunomodulatory antibodies in cancer. Nat. Rev. Cancer.

[21] Boruchov A.M., Heller G., Veri M.C., Bonvini E., Ravetch J.V., Young J.W. (2005). Activating and inhibitory IgG Fc receptors on human DCs mediate opposing functions. J. Clin. Investig..

[22] Dhodapkar K.M., Kaufman J.L., Ehlers M., Banerjee D.K., Bonvini E., Koenig S., Steinman R.M., Ravetch J.V., Dhodapkar M.V. (2005). Selective blockade of inhibitory Fcγ receptor enables human dendritic cell maturation with IL-12p70 production and immunity to antibody-coated tumor cells. Proc. Natl. Acad. Sci. USA.

[23] Garcia-Garijo A., Fajardo C.A., Gros A. (2019). Determinants for Neoantigen Identification. Front. Immunol..

[24] Lowery F.J., Krishna S., Yossef R., Parikh N.B., Chatani P.D., Zacharakis N., Parkhurst M.R., Levin N., Sindiri S., Sachs A. (2022). Molecular signatures of antitumor neoantigen-reactive T cells from metastatic human cancers. Science.

[25] Verdegaal E.M., de Miranda N.F., Visser M., Harryvan T., van Buuren M.M., Andersen R.S., Hadrup S.R., van der Minne C.E., Schotte R., Spits H. (2016). Neoantigen landscape dynamics during human melanoma-T cell interactions. Nature.

