# Supplementary material for: Targeted Delivery of Personalized Cancer Vaccines Based on Antibody–Antigen Complexes
Source: Vaccines (Basel). 2025 Mar 19;13(3):324. doi: 10.3390/vaccines13030324 (PMC11946472; doi:10.3390/vaccines13030324)
Supplement: Supplementary file 1 [file vaccines-13-00324-s001.zip › vaccines-3378280-supplementary.pdf]

Supporting information

Targeted delivery of personalized cancer vaccines based on antibody-antigen complexes

Yaling Zhang[1], Lingling Yan[1], He Sun[1], Ziyi Zhang[1], Fengyun Shen[2] * and Lele Sun[2] *

1 School of Life Sciences, Shanghai University, Shanghai 200444, China

2 Institute of Materiobiology, Department of Chemistry, College of Science, Shanghai University Shanghai, 200444 (China)

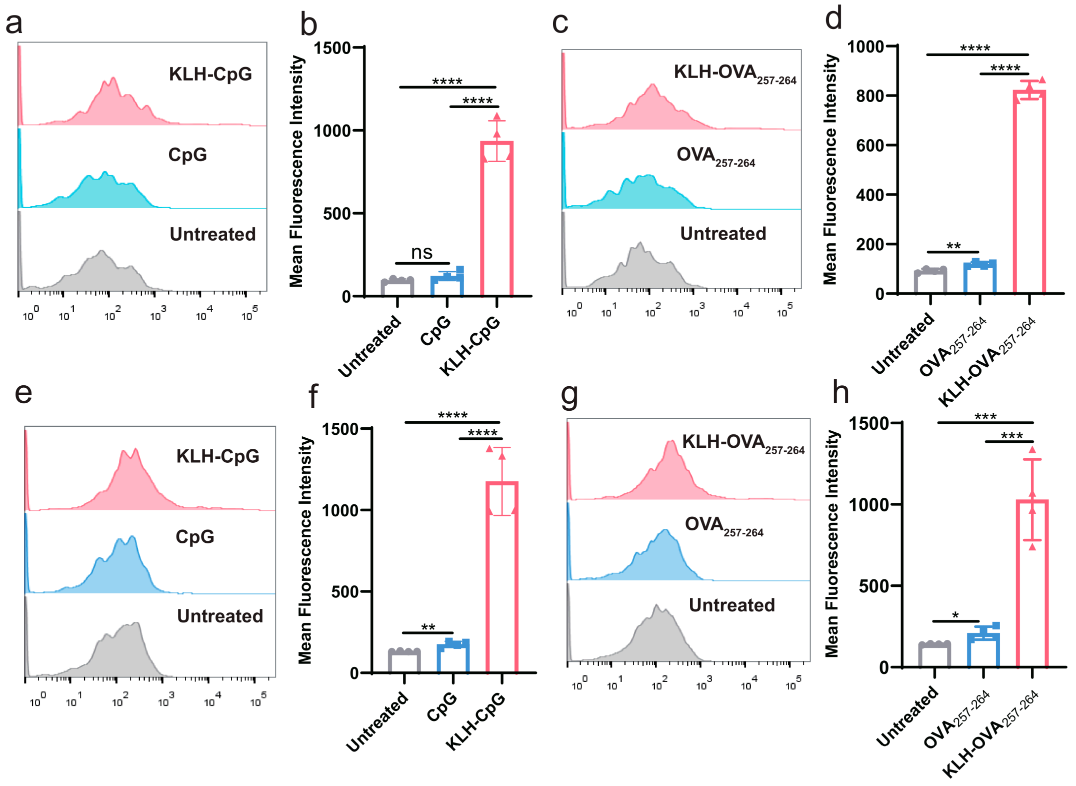

Figure S1. Conjugation with KLH carrier can enhance the uptake of CpG or $OVA_{257-264}$ by DCs and Mφ. (a-b) Flow cytometry analysis of the uptake of free CpG and KLH-CpG by $CD11c^+$ DCs in mouse spleen. (c-d) Flow cytometry analysis of the uptake of free $OVA_{257-264}$ and KLH-$OVA_{257-264}$ by $CD11c^+$ DCs in mouse spleen. (e-f) Flow cytometry analysis of the uptake of CpG and KLH-CpG by $F4/80^+$ Mφ in mouse spleen. (g-h) Flow cytometry analysis of the uptake of free $OVA_{257-264}$ and KLH-$OVA_{257-264}$ by $F4/80^+$ Mφ in mouse spleen. Data are shown as mean ± SD, n = 4. *$P < 0.05$, **$P < 0.01$, ***$P < 0.001$, and ****$P < 0.0001$.
